# Ultrastable cellulosome-adhesion complex tightens under load

**DOI:** 10.1038/ncomms6635

**Published:** 2014-12-08

**Authors:** Constantin Schoeler, Klara H. Malinowska, Rafael C. Bernardi, Lukas F. Milles, Markus A. Jobst, Ellis Durner, Wolfgang Ott, Daniel B. Fried, Edward A. Bayer, Klaus Schulten, Hermann E. Gaub, Michael A. Nash

**Affiliations:** 1 Lehrstuhl für Angewandte Physik and Center for Nanoscience, Ludwig-Maximilians-Universität, 80799 Munich, Germany; 2 Theoretical and Computational Biophysics Group, Beckman Institute for Advanced Science and Technology, University of Illinois at Urbana-Champaign, Urbana, Illinois 61801, USA; 3 Department of Biological Chemistry, The Weizmann Institute of Science, Rehovot 76100, Israel; 4 Department of Physics, University of Illinois at Urbana-Champaign, Urbana, Illinois 61801, USA

## Abstract

Challenging environments have guided nature in the development of ultrastable protein complexes. Specialized bacteria produce discrete multi-component protein networks called cellulosomes to effectively digest lignocellulosic biomass. While network assembly is enabled by protein interactions with commonplace affinities, we show that certain cellulosomal ligand–receptor interactions exhibit extreme resistance to applied force. Here, we characterize the ligand–receptor complex responsible for substrate anchoring in the *Ruminococcus flavefaciens* cellulosome using single-molecule force spectroscopy and steered molecular dynamics simulations. The complex withstands forces of 600–750 pN, making it one of the strongest bimolecular interactions reported, equivalent to half the mechanical strength of a covalent bond. Our findings demonstrate force activation and inter-domain stabilization of the complex, and suggest that certain network components serve as mechanical effectors for maintaining network integrity. This detailed understanding of cellulosomal network components may help in the development of biocatalysts for production of fuels and chemicals from renewable plant-derived biomass.

## Abstract (web summary)

Cellulosomes are cell surface enzyme complexes that digest lignocellulosic biomass. Here, Schoeler *et al.* characterize the strength of the ligand–receptor anchoring complex and find that it represents one of the strongest interactions known, and is strengthened under applied force by a catch bond mechanism.

## Graphical abstract

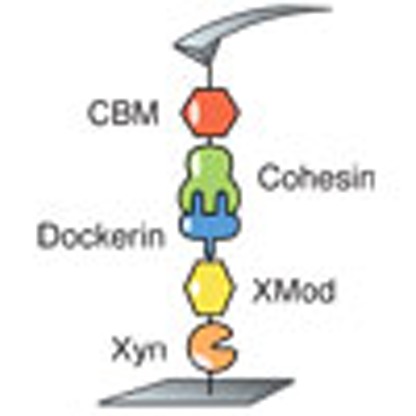

Cellulosomes are protein networks designed by nature to degrade lignocellulosic biomass^1^. These networks comprise intricate assemblies of conserved subunits including catalytic domains, scaffold proteins, carbohydrate binding modules (CBMs), cohesins (Cohs), dockerins (Docs) and X-modules (XMods) of unknown function. Coh:Doc pairs form complexes with high affinity and specificity^2^, and provide connectivity to a myriad of cellulosomal networks with varying Coh:Doc network topology^3^,^4^,^5^. The most intricate cellulosome known to date is produced by *Ruminococcus flavefaciens* (*R.f.*)^6^,^7^ and contains several primary and secondary scaffolds along with over 220 Doc-bearing protein subunits^8^.

The importance of cellulolytic enzymes for the production of renewable fuels and chemicals from biomass has highlighted an urgent need for improved fundamental understanding of how cellulosomal networks achieve their impressive catalytic activity^9^. Two of the mechanisms known to increase the catalytic activity of cellulosomes are proximity and targeting effects^10^. Proximity refers to the high local concentration of enzymes afforded by incorporation into nanoscale networks, while targeting refers to specific binding of cellulosomes to substrates. Protein scaffolds and CBM domains are both critical in this context as they mediate interactions between comparatively large bacterial cells and cellulose particles. As many cellulosomal habitats (for example, cow rumen) exhibit strong flow gradients, shear forces will accordingly stress bridging scaffold components mechanically *in vivo*. Protein modules located at stressed positions within these networks should therefore be preselected for high mechanostability. However, thus far very few studies on the mechanics of carbohydrate-active proteins or cellulosomal network components have been reported^11^.

In the present study we sought to identify cellulosomal network junctions with maximal mechanical stability. We chose an XMod-Doc:Coh complex responsible for maintaining bacterial adhesion to cellulose in the rumen. The complex links the *R. flavefaciens* cell wall to the cellulose substrate via two CBM domains located at the N-terminus of the CttA scaffold, as shown in Fig. 1a. The crystal structure of the complex solved by X-ray crystallography^12^ is shown in Fig. 1b. XMod-Doc tandem dyads such as this one are a common feature in cellulosomal networks. Bulk biochemical assays on XMod-Docs have demonstrated that XMods improve Doc solubility and increase biochemical affinity of Doc:Coh complex formation^13^. Crystallographic studies conducted on XMod-Doc:Coh complexes have revealed direct contacts between XMods and their adjacent Docs^12^,^14^. In addition, many XMods (for example, PDB 2B59, 1EHX, 3PDD) have high β-strand content and fold with N- and C-termini at opposite ends of the molecule, suggestive of robust mechanical clamp motifs at work^15^,^16^. These observations all suggest a mechanical role for XMods. Here we perform AFM single-molecule force spectroscopy experiments and steered molecular dynamics simulations to understand the mechanostability of the XMod-Doc:Coh cellulosomal ligand–receptor complex. We conclude that the high mechanostability we observe originates from molecular mechanisms, including stabilization of Doc by the adjacent XMod domain and catch bond behaviour that causes the complex to increase in contact area on application of force.

## Results and Discussion

### Single-molecule experiments

**Figure 1:**
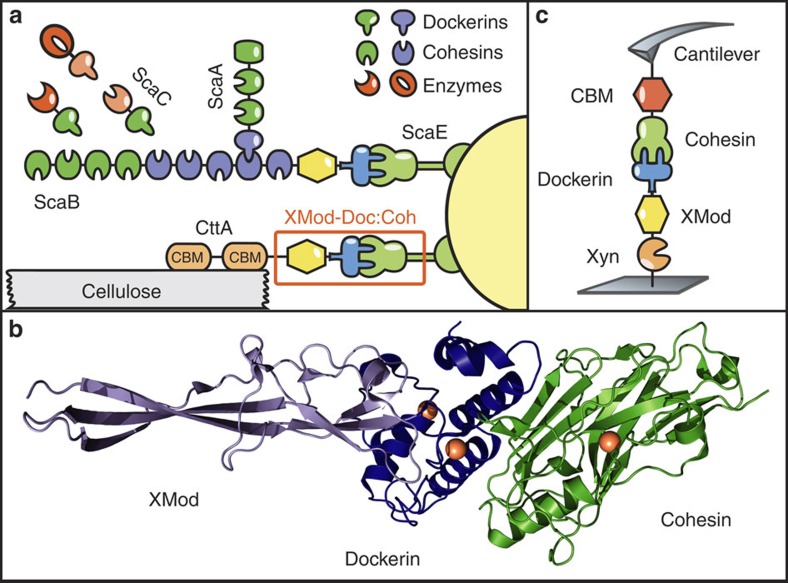
System overview. (**a**) Schematic of selected components of the *R. flavefaciens* cellulosome. The investigated XMod–Doc:Coh complex responsible for maintaining bacterial adhesion to cellulose is highlighted in orange. (**b**) Crystal structure of the XMod-Doc:Coh complex. Ca^2+^ ions are shown as orange spheres. (**c**) Depiction of experimental pulling configuration I, with Coh-CBM attached to the cantilever tip and Xyn–XMod–Doc attached to the glass surface.

We performed single-molecule force spectroscopy (SMFS) experiments with an atomic force miscroscope (AFM) to probe the mechanical dissociation of XMod-Doc:Coh. Xylanase (Xyn) and CBM fusion domains on the XMod-Doc and Coh modules, respectively, provided identifiable unfolding patterns permitting screening of large data sets of force-distance curves^17^,^18^,^19^. Engineered cysteines and/or peptide tags on the CBM and Xyn marker domains were used to covalently immobilize the binding partners in a site-specific manner to an AFM cantilever or cover glass via poly(ethylene glycol) (PEG) linkers. The pulling configuration with Coh-CBM immobilized on the cantilever is referred to as configuration I, as shown in Fig. 1c. The reverse configuration with Coh-CBM on the cover glass is referred to as configuration II. In a typical experimental run we collected about 50,000 force extension traces from a single cantilever. We note that the molecules immobilized on the cantilever and glass surfaces were stable over thousands of pulling cycles.

**Figure 2:**
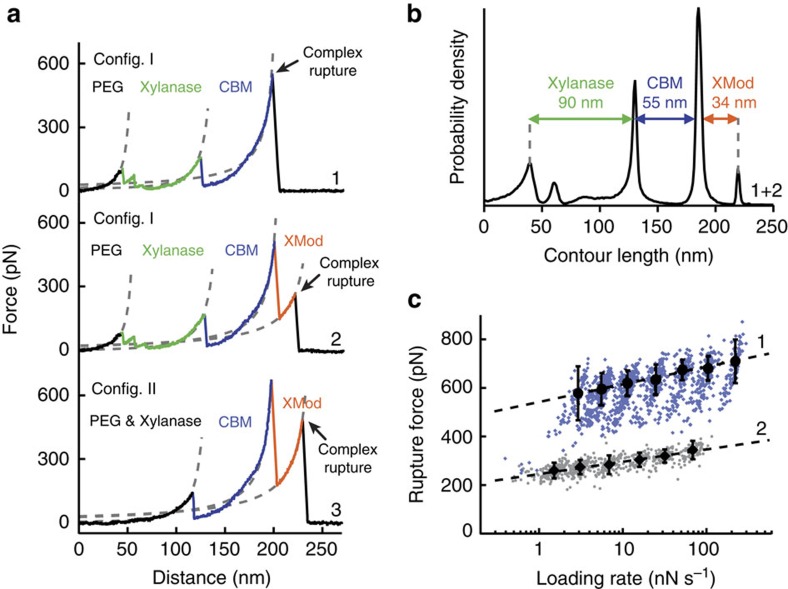
Experimental SMFS unfolding traces. (**a**) Unfolding fingerprints from pulling configuration I (curves 1 & 2) and configuration II (curve 3). The QM-FRC model (dashed lines) was used to estimate the contour lengths of the unfolded modules. (**b**) Contour length histogram obtained from 127 force extension traces (Config. I). The peak-to-peak increments correspond to Xyn, CBM and XMod amino-acid sequence lengths. (**c**) Dynamic force spectra for the final Doc:Coh complex rupture peaks obtained from 2,122 force-extension traces. The blue points show Doc:Coh ruptures that occurred with an intact XMod, while grey points show ruptures immediately following XMod unfolding. Black circles and diamonds represent the most probable rupture force/loading rate obtained by Gaussian fitting at each pulling speed. Error bars are ±1 s.d. Dashed lines are least square fits to the Bell-Evans model.

We sorted the data by first searching for contour length increments that matched our specific xylanase and CBM fingerprint domains. After identifying these specific traces (Fig. 2a), we measured the loading rate dependency of the final Doc:Coh ruptures based on bond history. To assign protein subdomains to the observed unfolding patterns, we transformed the data into contour length space using a freely rotating chain model with quantum mechanical corrections for peptide backbone stretching (QM-FRC, Supplementary Note 1, Supplementary Fig. 1)^20^,^21^. The fit parameter-free QM-FRC model describes protein stretching at forces >200 pN more accurately than the commonly used worm-like chain (WLC) model^20^,^22^. The resulting contour length histogram is shown in Fig. 2b. Peak-to-peak distances in the histogram represent contour length increments of unfolded protein domains. Assuming a length per stretched amino acid of 0.365 nm and accounting for the folded length of each subdomain, we compared the observed increments to the polypeptide lengths of individual subdomains of the Xyn-XMod-Doc and Coh-CBM fusion proteins. Details on contour length estimates and domain assignments are shown in Supplementary Table 1.

Unfolding patterns in configuration I showed PEG stretching followed by a three-peaked Xyn fingerprint (Fig. 1a, top trace, green), which added 90 nm of contour length to the system. Xyn unfolding was followed by CBM unfolding at ~150 pN with 55 nm of contour length added. Finally, the XMod-Doc:Coh complex dissociated at an ultra-high rupture force of ~600 pN. The loading rate dependence of the final rupture event for curves of subtype 1 is plotted in Fig. 2c (blue). The measured complex rupture force distributions are shown in Supplementary Fig. 2.

Less frequently (35–40% of traces) we observed a two-step dissociation process wherein the XMod unfolded before Doc:Coh rupture as shown in Fig. 2a (middle trace, orange). In these cases, the final dissociation exhibited a much lower rupture force (~300 pN) than the preceding XMod unfolding peak, indicating the strengthening effect of XMod was lost, and XMod was no longer able to protect the complex from dissociation at high force. The loading rate dependency of Doc:Coh rupture occurring immediately following XMod unfolding is shown in Fig. 2c (grey).

In configuration II (Fig. 2a, bottom trace), with the Xyn-XMod-Doc attached to the cantilever, the xylanase fingerprint was lost after the first few force extension traces acquired in the data set. This indicated the Xyn domain did not refold within the timescale of the experiment once unfolded, consistent with prior work^17^,^18^. CBM and XMod unfolding events were observed repeatedly throughout the series of acquired force traces in both configurations I and II, indicating these domains were able to refold while attached to the cantilever over the course of the experiment.

We employed the Bell-Evans model^23^ (Supplementary Note 2) to analyse the final rupture of the complex through the effective distance to the transition state (Δ*x*) and the natural off-rate (*k*_off_). The fits to the model yielded values of Δ*x*=0.13 nm and *k*_off_=7.3 × 10^−7^s^−1^ for an intact XMod, and Δ*x*=0.19 nm and *k*_off_=4.7 × 10^−4^ s^−1^ for the ‘shielded’ rupture following XMod unfolding (Fig. 2c). These values indicate that the distance to the transition state is increased following XMod unfolding, reflecting an overall softening of the binding interface. Distances to the transition state observed for other ligand–receptor pairs are typically on the order of ~0.7 nm (ref. 17). The extremely short Δ*x* of 0.13 nm observed here suggests that mechanical unbinding for this complex is highly coordinated. We further analysed the unfolding of XMod in the Bell-Evans picture and found values of Δ*x*=0.15 and *k*_off_=2.6 × 10^−6^s^−1^. The loading rate dependence for this unfolding event is shown in Supplementary Fig. 3.

The exceptionally high rupture forces measured experimentally (Fig. 2) are hugely disproportionate to the XMod-Doc:Coh biochemical affinity, which at *K*_D_~20 nM (ref. 12) is comparable to typical antibody–antigen interactions. Antibody–antigen interactions, however, will rupture at only ~60 pN at similar loading rates^24^, while bimolecular complexes found in muscle exposed to mechanical loading *in vivo* will rupture at ~140 pN (ref. 25). Trimeric titin–telethonin complexes also found in muscle exhibit unfolding forces around 700 pN (ref. 26), while Ig domains from cardiac titin will unfold at ~200 pN (ref. 27). The XMod-Doc:Coh ruptures reported here fell in a range from 600 to 750 pN at loading rates ranging from 10 to 100 nN s^−1^. At around half the rupture force of a covalent gold-thiol bond^28^, these bimolecular protein rupture forces are, to the best of our knowledge, among the highest of their kind ever reported. The covalent bonds in this system are primarily peptide bonds in the proteins and C-C and C-O bonds in the PEG linkers. These are significantly more mechanically stable than the quoted gold-thiol bond rupture force (~1.2 nN) (ref. 29) and fall in a rupture force range >2.5 nN at similar loading rates. Therefore, breakage of covalent linkages under our experimental conditions is highly unlikely. We note that the high mechanostability observed here is not the result of fusing the proteins to the CBM or Xyn domains. The covalent linkages and pulling geometry are consistent with the wild-type complex and its dissociation pathway. *In vivo*, the Coh is anchored to the peptidoglycan cell wall through its C-terminal sortase motif. The XMod–Doc is attached to the cellulose substrate through two N-terminal CBM domains. By pulling the XMod–Doc through an N-terminal Xyn fusion domain, and the Coh through a C-terminal CBM, we established an experimental pulling geometry that matches loading of the complex *in vivo*. This pulling geometry was also used in all simulations. The discontinuity between its commonplace biochemical affinity and remarkable resistance to applied force illustrates how this complex is primed for mechanical stability and highlights differences in the unbinding pathway between dissociation at equilibrium and dissociation induced mechanically along a defined pulling coordinate.

### Steered molecular dynamics

To elucidate the molecular mechanisms at play that enable this extreme mechanostability, we carried out all-atom steered molecular dynamics (SMD) simulations. The Xyn and CBM domains were not modelled to keep the simulated system small and reduce the usage of computational resources. This approximation was reasonable as we have no indication that these domains significantly affect the XMod–Doc:Coh binding strength^30^. After equilibrating the crystal structure^12^, the N-terminus of XMod–Doc was harmonically restrained while the C-terminus of Coh was pulled away at constant speed. The force applied to the harmonic pulling spring was stored at each time step. We tested pulling speeds of 0.25, 0.625 and 1.25 Å ns^−1^, and note that the slowest simulated pulling speed was ~4,000 times faster than our fastest experimental pulling speed of 6.4 μm s^−1^. This difference is considered not to affect the force profile, but it is known to account for the scale difference in force measured by SMD and AFM^31^,^32^.

**Figure 3:**
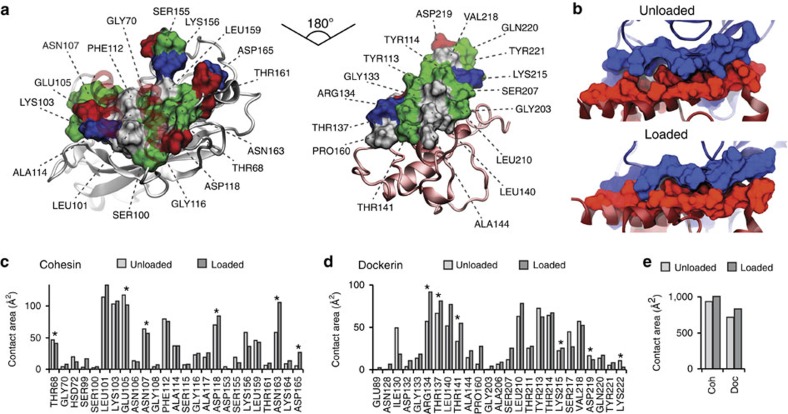
Analysis of binding interface and catch bond mechanism from SMD. (**a**) Surface plots for the main interacting residues of Coh (left) and Doc (right). Hydrophobic residues are shown in grey, polar residues in green, and negative and positive residues in red and blue, respectively. Both Coh and Doc exhibit a hydrophobic patch in the centre of the binding surface that is surrounded by polar and charged residues. (**b**) Rearrangement of binding residues of Coh (blue) and Doc (red) under force. Following mechanical loading, an interdigitated complex is formed that resembles teeth of a zipper. (**c**,**d**) Surface contact area of interacting residues of Coh (c) and Doc (d) in the absence and presence of force. Residues forming prevalent hydrogen bonds are indicated with stars. (**e**) Total contact surface area of Coh and Doc in unloaded and loaded conformations.

SMD results showed the force increased with distance until the complex ruptured for all simulations. At the slowest pulling speed of 0.25 Å ns^−1^ the rupture occurred at a peak force of ~900 pN, as shown in Supplementary Fig. 4 and Supplementary Movie 1. We analysed the progression and prevalence of hydrogen bonded contacts between the XMod–Doc and Coh domains to identify key residues in contact throughout the entire rupture process and particularly immediately before rupture. These residues are presented in Fig. 3a,c,d and Supplementary Figs 5,6. The simulation results clearly reproduced key hydrogen bonding contacts previously identified^12^ as important for Doc:Coh recognition (Supplementary Fig. 5).

The main interacting residues are shown in Fig. 3a,b. Both Coh and Doc exhibit a binding interface consisting of a hydrophobic centre (grey) surrounded by a ring of polar (green) and charged residues (blue, positive; red, negative). This residue pattern suggests the hydrophilic side chains protect the interior hydrophobic core from attack by water molecules, compensating for the flat binding interface that lacks a deep pocket. The geometry suggests a penalty to unbinding that stabilizes the bound state. Further, we analysed the contact surface areas of interacting residues (Fig. 3b–e). The total contact area was found to increase due to rearrangement of the interacting residues when the complex is mechanically stressed, as shown in Fig. 3e and Supplementary Movie 2. Doc residues in the simulated binding interface clamped down on Coh residues upon mechanical loading, resulting in increased stability and decreased accessibility of water into the hydrophobic core of the bound complex (Fig. 3b). These results suggest that a catch bond mechanism is responsible for the remarkable stability^33^ under force and provide a molecular mechanism which the XMod–Doc:Coh complex uses to summon mechanical strength when needed, while still allowing relatively fast assembly and disassembly of the complex at equilibrium. The residues that increase most in contact area (Fig. 3c,d) present promising candidates for future mutagenesis studies.

**Figure 4:**
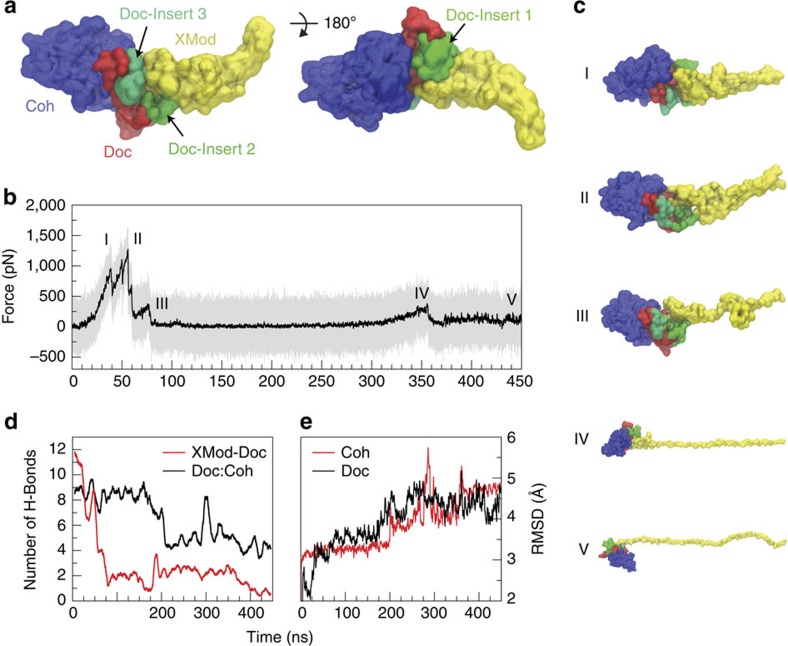
SMD shows unfolding of XMod destabilizes Doc:Coh binding interface. XMod was unfolded by moving the harmonic restraint to the C terminus of XMod while the N terminus was moved at 0.625 Å ns^−1^. (**a**) Surface representation of XMod-Doc:Coh complex with Doc insert sequences. Coh is shown in blue, Doc in red and green (inserts), and XMod in yellow. (**b**) Force time trace of XMod unfolding. The domain starts to unfold in several substeps starting at ~400 pN. Snapshots at different time steps are labelled I-V and are shown in (**c**). Steps IV and V are shown at smaller scale. (**d**) Average number of hydrogen bonds between Doc:Coh (black) and XMod-Doc (red). XMod-Doc contact is dominated by the insert sequences 1–3. (**e**) Root mean squared deviation (RMSD) of Doc (black) and Coh (red).

Among the 223 Doc sequences from *R. flavefaciens*, six subfamilies have been explicitly identified using bioinformatics approaches^8^. The XMod–Doc investigated here belongs to the 40-member Doc family 4a. A conserved feature of these Doc modules is the presence of three sequence inserts that interrupt the conserved duplicated F-hand motif Doc structure. In our system, these Doc sequence inserts make direct contacts with XMod in the crystallized complex (Fig. 1) and suggest an interaction between XMod and Doc that could potentially propagate to the Doc:Coh binding interface. To test this, an independent simulation was performed to unfold XMod (Fig. 4). The harmonic restraint was moved to the C-terminus of XMod so that force was applied from the N- to C-terminus of XMod only, while leaving Doc and Coh unrestrained. The results (Fig. 4b) showed XMod unfolded at forces slightly higher than but similar to the XMod–Doc:Coh complex rupture force determined from the standard simulation at the same pulling speed. This suggested XMod unfolding before Doc:Coh rupture was not probable, but could be observed on occasion due to the stochastic nature of domain unfolding. This was consistent with experiments where XMod unfolding was observed in ~35–40% of traces. Furthermore, analysis of the H-bonding between Doc and XMod (Fig. 4d, red) indicated loss of contact as XMod unfolded, dominated by contact loss between the three Doc insert sequences and XMod. Interestingly, XMod unfolding clearly led to a decrease in H-bonding between Doc and Coh at a later stage (~200 ns) well after XMod had lost most of its contact with Doc, even though no force was being applied across the Doc:Coh binding interface. This provided evidence for direct stabilization of the Doc:Coh binding interface by XMod. As shown in Fig. 4e, the root mean squared deviation (RMSD) of Doc increased throughout the simulation as XMod unfolded. Coh RMSD remained stable until it started to lose H-bonds with Doc. Taken together this suggests that, as XMod unfolded, Coh and Doc became more mobile and lost interaction strength, potentially explaining the increase in Δ*x* from 0.13 to 0.19 nm on unfolding of XMod in the experimental data sets. Apparently the XMod is able to directly stabilize the Doc:Coh interface, presumably through contact with Doc insert sequences that then propagate this stabilizing effect to the Doc:Coh binding interface.

In summary, we investigated an ultrastable XMod-Doc:Coh complex involved in bacterial adhesion to cellulose. While previously the role of XMod functioning in tandem XMod-Doc dyads was unclear^12^,^14^, we show that XMod serves as a mechanical stabilizer and force-shielding effector subdomain in the ultrastable ligand–receptor complex. The Doc:Coh complex presented here exhibits one of the most mechanically robust protein–protein interactions reported thus far, and points towards new mechanically stable artificial multi-component biocatalysts for industrial applications, including production of second-generation biofuels.

## Methods

### Site-directed mutagenesis

Site-directed mutagenesis of *R. flavefaciens* strain FD1 chimeric cellulosomal proteins. A pET28a vector containing the previously cloned *R. flavefaciens* CohE from ScaE fused to cellulose-binding module 3a (CBM3a) from *C. thermocellum*, and a pET28a vector containing the previously cloned *R. flavefaciens* XMod-Doc from the CttA scaffoldin fused to the XynT6 xylanase from *Geobacillus stearothermophilus*^12^ were subjected to QuikChange mutagenesis^34^ to install the following mutations: A2C in the CBM and T129C in the xylanase, respectively.

For the construction of the native configuration of the CohE-CBM A2C fusion protein Gibson assembly^35^ was used. For further analysis CohE-CBM A2C was modified with a QuikChange PCR^36^ to replace the two cysteins (C2 and C63) in the protein with alanine and serine (C2A and C63S). All mutagenesis products were confirmed by DNA sequencing analysis.

The XynT6-XDoc T129C was constructed using the following primers:

5′-acaaggaaggtaagccaatggttaatgaatgcgatccagtgaaacgtgaac-3′

5′-gttcacgtttcactggatcgcattcattaaccattggcttaccttccttgt-3′

The CBM-CohE A2C was constructed using the following primers:

5′-ttaactttaagaaggagatataccatgtgcaatacaccggtatcaggcaatttgaag-3′

5′-cttcaaattgcctgataccggtgtattgcacatggtatatctccttcttaaagttaa-3′

The CohE-CBM C2A C63S was constructed using the following phosphorylated primers:

5′-ccgaatgccatggccaatacaccgg-3′

5′-cagaccttctggagtgaccatgctgc-3′

### Expression and purification of Xyn-XMod-Doc

The T129C Xyn-XMod-Doc protein was expressed in *E. coli* BL21 cells in kanamycin-containing media that also contained 2 mM calcium chloride, overnight at 16 °C. After harvesting, cells were lysed using sonication. The lysate was then pelleted, and the supernatant fluids were applied to a Ni-NTA column and washed with tris-buffered saline (TBS) buffer containing 20 mM imidazole and 2 mM calcium chloride. The bound protein was eluted using TBS buffer containing 250 mM imidazole and 2 mM calcium chloride. The solution was dialysed with TBS to remove the imidazole, and then concentrated using an Amicon centrifugal filter device and stored in 50% (v/v) glycerol at −20 °C. The concentrations of the protein stock solutions were determined to be ~5 mg ml^−1^ by absorption spectrophotometry.

### Expression and purification of Coh-CBM

The Coh-CBM C2A, C63S fusion protein was expressed in *E. coli* BL21(DE3) RIPL in kanamycin and chloramphenicol containing ZYM-5052 media^37^ overnight at 22 °C. After harvesting, cells were lysed using sonication. The lysate was then pelleted, and the supernatant fluids were applied to a Ni-NTA column and washed with TBS buffer. The bound protein was eluted using TBS buffer containing 200 mM imidazole. Imidazole was removed with a polyacrylamide gravity flow column. The protein solution was concentrated with an Amicon centrifugal filter device and stored in 50% (v/v) glycerol at −80 °C. The concentrations of the protein stock solutions were determined to be ~5 mg ml^−1^ by absorption spectrophotometry.

### Sample preparation

In sample preparation and single-molecule measurements calcium supplemented TBS buffer (Ca-TBS) was used (25 mM TRIS, 72 mM NaCl, 1 mM CaCl_2_, pH 7.2). Cantilevers and cover glasses were functionalized according to previously published protocols^18^,^38^. In brief, cantilevers and cover glasses were cleaned by UV-ozone treatment and piranha solution, respectively. Levers and glasses were silanized using (3-aminopropyl)-dimethyl-ethoxysilane (APDMES) to introduce surface amine groups. Amine groups on the cantilevers and cover glasses were subsequently conjugated to a 5 kDa NHS-PEG-Mal linker in sodium borate buffer. Disulfide-linked dimers of the Xyn-XMod-Doc proteins were reduced for 2 h at room temperature using a TCEP disulfide reducing bead slurry. The protein/bead mixture was rinsed with Ca-TBS measurement buffer, centrifuged at 850 r.c.f. for 3 min, and the supernatant was collected with a micropipette. Reduced proteins were diluted with measurement buffer (1:3 (v/v) for cantilevers, and 1:1 (v/v) for cover glasses), and applied to PEGylated cantilevers and cover glasses for 1 h. Both cantilevers and cover glasses were then rinsed with Ca-TBS to remove unbound proteins and stored under Ca-TBS before force spectroscopy measurements. Site-specific immobilization of the Coh-CBM-ybbR fusion proteins to previously PEGylated cantilevers or coverglasses was carried out according to previously published protocols^39^. In brief, PEGylated cantilevers or coverglasses were incubated with Coenzyme A (CoA) (20 mM) stored in coupling buffer (50 mM sodium phosphate, 50 mM NaCl, 10 mM EDTA, pH 7.2) for 1 h at room temperature. Levers or surfaces were then rinsed with Ca-TBS to remove unbound CoA. Coh-CBM-ybbR fusion proteins were then covalently linked to the CoA surfaces or levers by incubating with Sfp phosphopantetheinyl transferase for 2 h at room 37°. Finally, surfaces or levers were subjected to a final rinse with Ca-TBS and stored under Ca-TBS before measurement.

### Single-molecule force spectroscopy measurements

SMFS measurements were performed on a custom built AFM^40^ controlled by an MFP-3D controller from Asylum Research running custom written Igor Pro (Wavemetrics) software. Cantilever spring constants were calibrated using the thermal noise/equipartition method^41^. The cantilever was brought into contact with the surface and withdrawn at constant speed ranging from 0.2 to 6.4 μm s^−1^. An x-y stage was actuated after each force-extension trace to expose the molecules on the cantilever to a new molecule at a different surface location with each trace. Typically 20,000–50,000 force-extension curves were obtained with a single cantilever in an experimental run of 18–24 h. A low molecular density on the surface was used to avoid formation of multiple bonds. While the raw data sets contained a majority of unusable curves due to lack of interactions or nonspecific adhesion of molecules to the cantilever tip, select curves showed single-molecule interactions. We filtered the data using a combination of automated data processing and manual classification by searching for contour length increments that matched the lengths of our specific protein fingerprint domains: Xyn (~89 nm) and CBM (~56 nm). After identifying these specific traces, we measured the loading rate dependency of the final Doc:Coh ruptures based on bond history.

### Data analysis

Data were analysed using previously published protocols^17^,^18^,^22^. Force extension traces were transformed into contour length space using the QM-FRC model with bonds of length *b*=0.11 nm connected by a fixed angle *γ*=41° and and assembled into barrier position histograms using cross-correlation. Detailed description of the contour length transformation can be found in Supplementary Note 1 and Supplementary Fig. 1.

For the loading rate analysis, the loading rate at the point of rupture was extracted by applying a line fit to the force vs time trace in the immediate vicinity before the rupture peak. The loading rate was determined from the slope of the fit. The most probable rupture forces and loading rates were determined by applying Gaussian fits to histograms of rupture forces and loading rates at each pulling speed.

### Molecular dynamics simulations

The structure of the XMod-Doc:Coh complex had been solved by means of X-ray crystallography at 1.97 Å resolution and is available at the protein data bank (PDB:4IU3). A protonation analysis performed in VMD^42^ did not suggest any extra protonation and all the amino-acid residues were simulated with standard protonation states. The system was then solvated, keeping also the water molecules present in the crystal structure, and the net charge of the protein and the calcium ions was neutralized using sodium atoms as counter ions, which were randomly arranged in the solvent. Two other systems, based on the aforementioned one, were created using a similar salt concentration to the one used in the experiments (75 mM of NaCl). This additional salt caused little or no change in SMD results. The overall number of atoms included in MD simulations varied from 300,000 in the majority of the simulations to 580,000 for the unfolding of the X-Mod.

The MD simulations in the present study were performed employing the NAMD molecular dynamics package^43^,^44^. The CHARMM36 force field^45^,^46^ along with the TIP3 water model^47^ was used to describe all systems. The simulations were done assuming periodic boundary conditions in the NpT ensemble with temperature maintained at 300 K using Langevin dynamics for pressure, kept at 1 bar, and temperature coupling. A distance cut-off of 11.0 Å was applied to short-range, non-bonded interactions, whereas long-range electrostatic interactions were treated using the particle-mesh Ewald (PME)^48^ method. The equations of motion were integrated using the r-RESPA multiple time step scheme^44^ to update the van der Waals interactions every two steps and electrostatic interactions every four steps. The time step of integration was chosen to be 2 fs for all simulations performed. Before the MD simulations all the systems were submitted to an energy minimization protocol for 1,000 steps. The first two nanoseconds of the simulations served to equilibrate systems before the production runs that varied from 40 to 450 ns in the 10 different simulations that were carried out. The equilibration step consisted of 500 ps of simulation where the protein backbone was restrained and 1.5 ns where the system was completely free and no restriction or force was applied. During the equilibration the initial temperature was set to zero and was constantly increased by 1 K every 100 MD steps until the desired temperature (300 K) was reached.

To characterize the coupling between Doc and Coh, we performed SMD simulations^49^ of constant velocity stretching (SMD-CV protocol) employing three different pulling speeds: 1.25, 0.625 and 0.25 Å ns^−1^. In all simulations, SMD was employed by restraining the position of one end of the XMod-Doc domain harmonically (center of mass of ASN5), and moving a second restraint point, at the end of the Coh domain (center of mass of GLY210), with constant velocity in the desired direction. The procedure is equivalent to attaching one end of a harmonic spring to the end of a domain and pulling on the other end of the spring. The force applied to the harmonic spring is then monitored during the time of the molecular dynamics simulation. The pulling point was moved with constant velocity along the *z*-axis and due to the single anchoring point and the single pulling point the system is quickly aligned along the *z*-axis. Owing to the flexibility of the linkers, this approach reproduces the experimental set-up. All analyses of MD trajectories were carried out employing VMD^42^ and its plug-ins. Secondary structures were assigned using the Timeline plug-in, which employs STRIDE criteria^50^. Hydrogen bonds were assigned based on two geometric criteria for every trajectory frame saved: first, distances between acceptor and hydrogen should be <3.5 Å; second, the angle between hydrogen-donor-acceptor should be <30°. Surface contact areas of interacting residues were calculated employing Volarea^51^ implemented in VMD. The area is calculated using a probe radius defined as an *in silico* rolling spherical probe that is screened around the area of Doc exposed to Coh and also Coh area exposed to Doc.

## Additional information

**Accession codes:** Plasmids used in this study are available through Addgene ( https://www.addgene.org) under the following accession codes: Xylanase-Xmodule-Dockerin: 60865; Cohesin-CBM: 60866.

**How to cite this article:** Schoeler, C. *et al.* Ultrastable cellulosome-adhesion complex tightens under load. *Nat. Commun.* 5:5635 doi: 10.1038/ncomms6635 (2014).

## Supplementary Material

Supplementary Information Supplementary Figures 1-6, Supplementary Table 1, Supplementary Note 1, Supplementary Methods and Supplementary References

Supplementary Movie 1 Steered molecular dynamics simulation of constant velocity stretching of the XMod-Doc:Coh complex at a pulling speed of 0.25 Å ns-1. Doc is highlighted in blue, Coh in red and XMod in yellow. Orange and green spheres represent harmonically restrained N-terminus of Xmod and C-terminus of Coh that was moved with constant velocity, respectively.

Supplementary Movie 2 Close-up to the binding interface during steered molecular dynamics simulation of constant velocity stretching of XMod-Doc:Coh complex at a pulling speed of 0.25 Å ns-1. Surfaces of the interacting residues are highlighted in blue (Coh) and red (Doc).
