# Supplementary material for: Ultrastable cellulosome-adhesion complex tightens under load
Source: Nat Commun. 2014 Dec 8;5:5635. doi: 10.1038/ncomms6635 (PMC4266597; doi:10.1038/ncomms6635)
Supplement: Supplementary Information — Supplementary Figures 1-6, Supplementary Table 1, Supplementary Note 1, Supplementary Methods and Supplementary References [file ncomms6635-s1.pdf]

Supplementary Figures

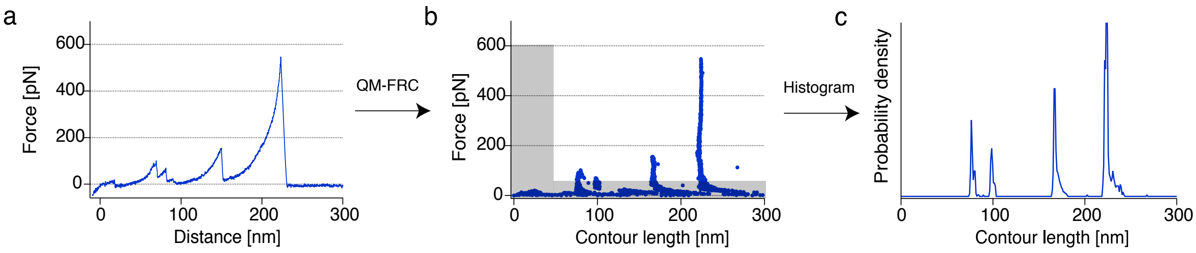

Supplementary Fig. 1: Assembly of contour length histograms. **a** Force-extension traces are transformed into contour length space using a QM-corrected FRC model with parameters $\gamma = 41°$, and $b = 0.11\,\mathrm{nm}$. **b** In force-contour length space, force and contour length thresholds are applied and the data are histogrammed with a bin width of $1\,\mathrm{nm}$ to obtain the histogram in **c**. To obtain a master histogram, individual histograms reflecting a specific unfolding pathway are cross-correlated and aligned by offsetting by the maximum correlation value.

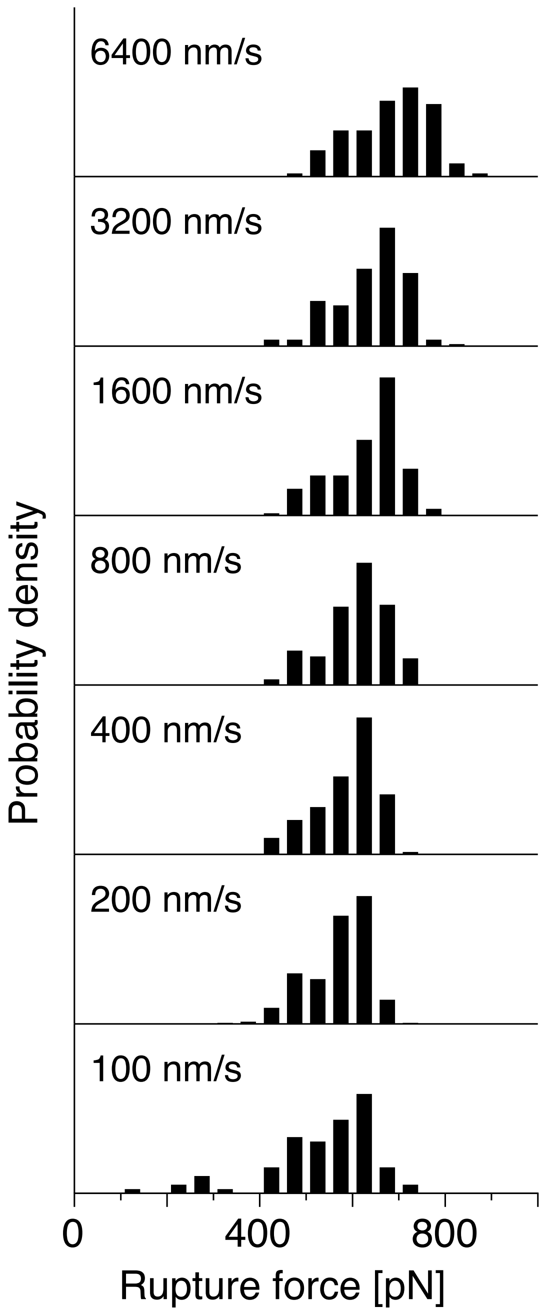

Supplementary Fig. 2: Complex rupture force histograms for pulling speeds ranging from 100 $\mathsf{nm\,s^{-1}}$ to 6400 $\mathsf{nm\,s^{-1}}$. Pulling speeds are indicated next to the histograms. Only traces with an intact XMod were taken into account (no XMod unfolding observed, corresponding to Fig. 2, trace 1). At the slowest pulling speed data suggest the presence of a lower rupture force population.

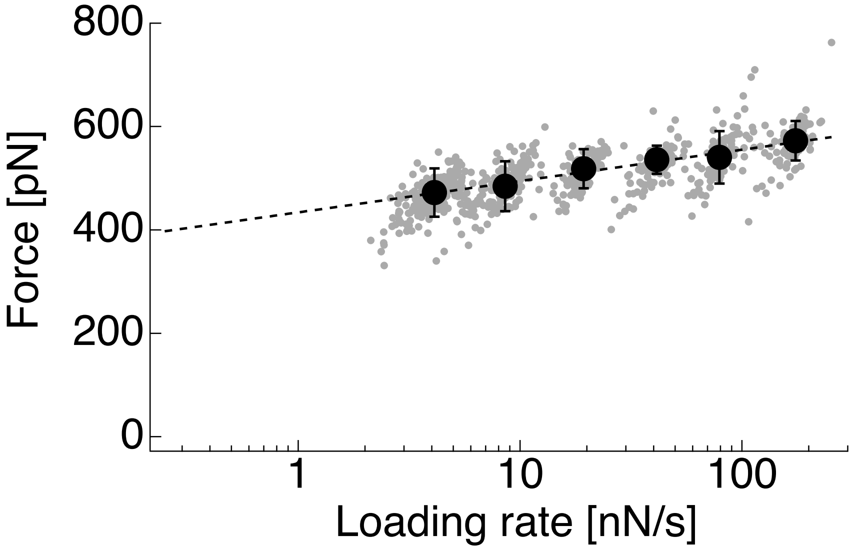

Supplementary Fig. 3: Dynamic force spectrum for XMod unfolding obtained from 654 force-extension traces. The gray points show single XMod unfolding events. Black circles represent the most probable rupture forces and loading rates obtained by Gaussian fitting at each pulling speed. Error bars are ± 1 standard deviation. The dashed line is a least squares fit to the Bell-Evans model that yielded $\Delta x = 0.15\,\mathrm{nm}$ and $k_{off} = 2.6 \times 10^{-6}\,\mathrm{s}^{-1}$.

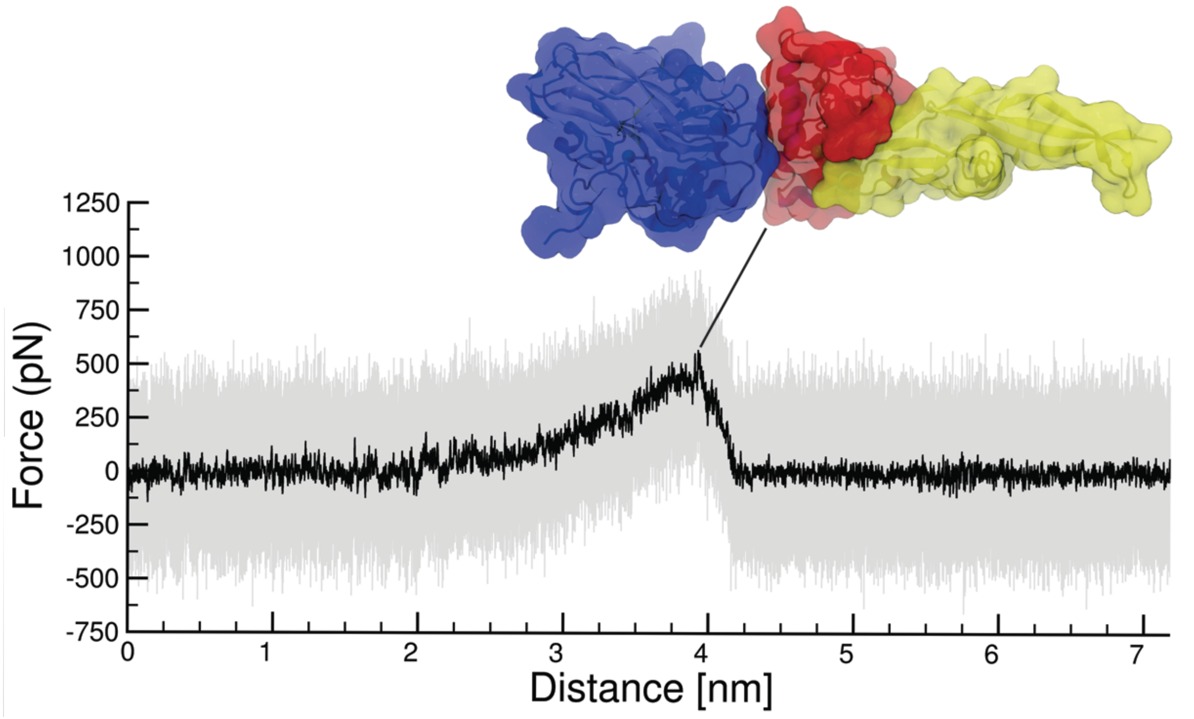

Supplementary Fig. 4: Force distance trace obtained by SMD at a pulling speed of 0.25 Å ns^{-1}. Force values at each time step are shown in gray, with average force calculated every 200 ps in black. The inset is a snapshot of the XMod-Doc:Coh complex immediately prior to rupture. XMod is shown in yellow, Doc in red and Coh in blue.

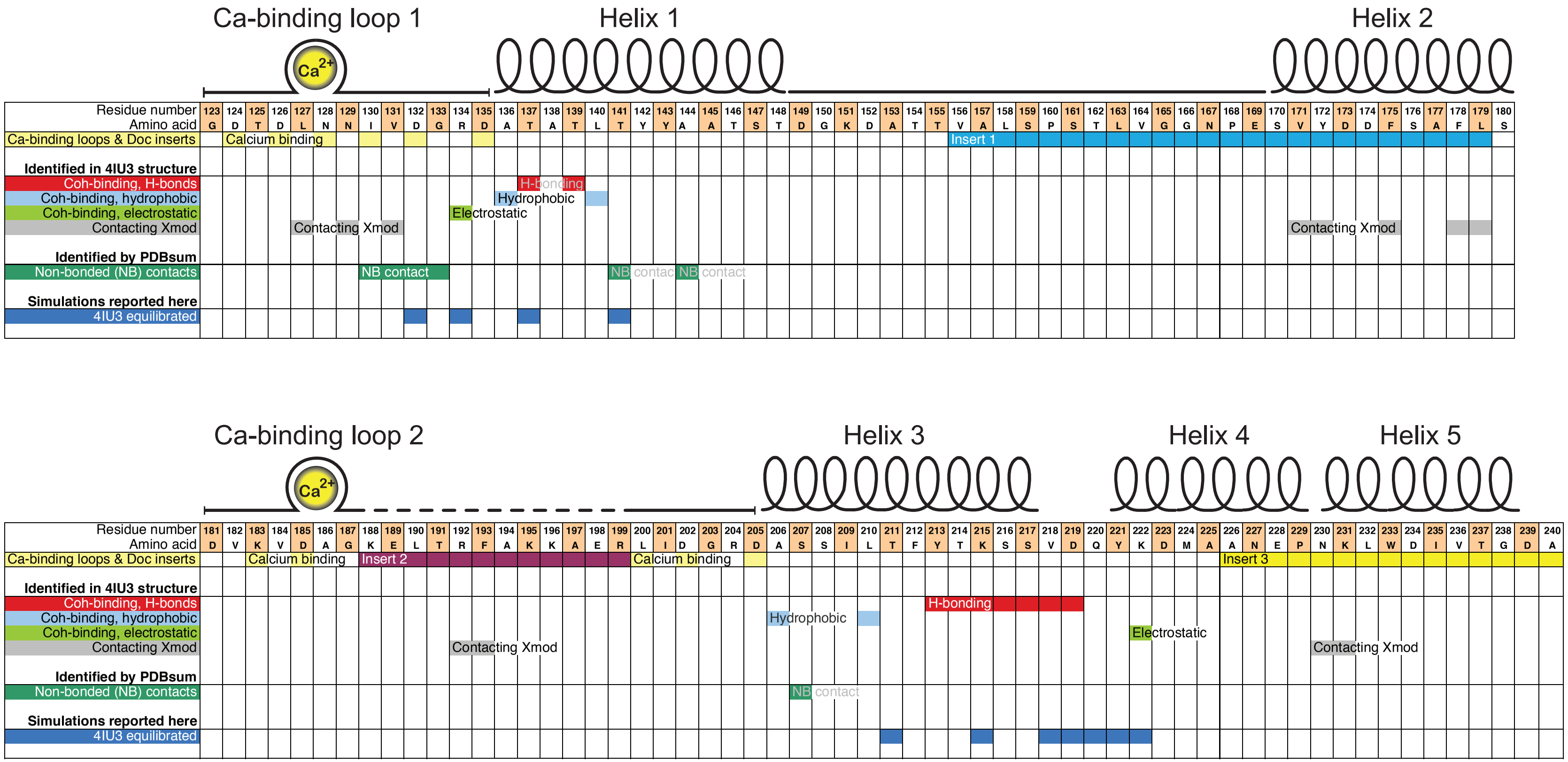

Supplementary Fig. 5: Amino acid sequence and secondary structure elements of the *Ruminococcus flavefaciens* dockerin module. The table includes interactions with the Coh and XMod domains identified in the PDB code 4IU3 crystal structure, PDBsum[1] and MD simulations.

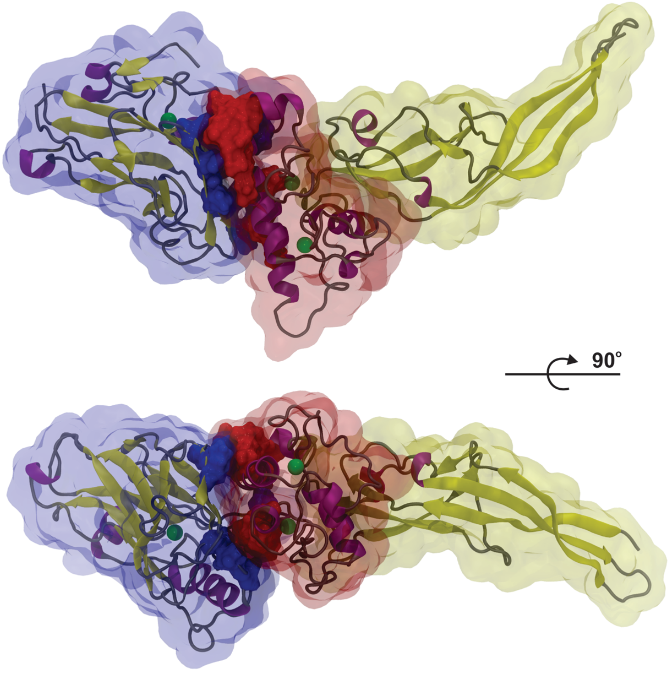

Donor	Acceptor	Prevalence
LYS215-S	GLU105-S	87%
ASN107-S	ASP219-S	48%
ASP219-B	ASN107-S	39%
THR141-S	ASP118-S	31%
LYS156-S	ASP132-S	22%

Supplementary Fig. 6: Hydrogen bond contacts between XMod-Doc (yellow and red surface, respectively) and Coh (blue surface). The residues that have hydrogen bonds lasting for more than 10% of the simulation time are represented in a glossy surface. In the bottom of the figure the five most prevalent hydrogen bond interactions are presented. The letter S or B indicate if the respective interaction is made by the amino acid side chain or backbone.

Supplementary Tables

Module	Xylanase	CBM	X-module	Cohesin	Dockerin
No. amino acids, N_A	260 (378)	159	117	205	119
Folded length, L_F [nm]	6	2	7	2	2
Expected increment, ΔL_E [nm]	89	56	36	72	42
Observed increment, [nm]	90 ± 4	55 ± 3	34 ± 2	–	–

Supplementary Table 1: Domain assignment of observed contour length increments. The expected contour length increment (ΔL_E) for each protein domain was calculated according to $\Delta L_E = N_A \cdot 0.365\,\mathrm{nm} - L_F$, where L_F is the folded length, N_A is the number of amino acids, and $0.365\,\mathrm{nm}^2$ is the length per stretched amino acid. L_F was measured for Xyn, CBM, and XDoc:Coh from PDB structures 1R85, 1NBC, and 4IU3, respectively. For the Xyn domain, only amino acids located C-terminal of the C129 mutation which served as attachment point are considered. Errors for the observed increments were determined from Gaussian fits to the combined contour length histogram shown in Fig. 2b.

Supplementary Notes

Supplementary Note 1: QM-FRC Model for Polymer Elasticity

The freely rotating chain model[3] considers bonds of length b, connected by a fixed angle γ. The torsional angles are not restricted. The stretching behavior in the FRC picture is given by

$$\frac{x}{L} = \begin{cases} \frac{Fa}{3k_BT} & \text{for} \quad \frac{Fb}{k_BT} < \frac{b}{p} \\ 1 - \left(\frac{4Fp}{k_BT}\right)^{-\frac{1}{2}} & \text{for} \quad \frac{b}{p} < \frac{Fb}{k_BT} < \frac{p}{b} \\ 1 - \left(\frac{cFb}{k_BT}\right)^{-1} & \text{for} \quad \frac{p}{b} < \frac{Fb}{k_BT} \end{cases} \tag{1}$$

where $a = b\frac{1+\cos\gamma}{(1-\cos\gamma)\cos\frac{\gamma}{2}}$ is the Kuhn length, and $p = b\frac{\cos\frac{\gamma}{2}}{|\ln(\cos\gamma)|}$ is the effective persistence length in the FRC picture.

To account for backbone elasticity of the polypeptide chain at high force, quantum mechanical *ab-initio* calculations can be used to obtain the unloaded contour length at zero force. A polynomial approximation to these calculations can be used to obtain the unloaded contour length at zero force L_0:

$$F = \gamma_1\left(\frac{L}{L_0} - 1\right) + \gamma_2\left(\frac{L}{L_0} - 1\right)^2 \tag{2}$$

where the $\gamma_1 = 27.4\,\mathrm{nN}$, and $\gamma_2 = 109.8\,\mathrm{nN}$ are the elastic coefficients reported for polypeptides[4].

Supplementary Note 2: Bell-Evans Model for Mechanically Induced Receptor Ligand Dissociation

The Bell-Evans model was used to estimate the distance to the transition state (Δx) and the natural off-rate (k_{off}) of individual rupture events:

$$\langle F \rangle = \frac{k_BT}{\Delta x}\ln\frac{\Delta x \cdot \dot{F}}{k_{off}k_BT} \tag{3}$$

where k_B is Boltzmann's constant, T is the temperature and $\dot{F}$ is the loading rate at the point of rupture.

Supplementary Methods

Materials

Silicon nitride cantilevers (Biolever mini, BL-AC40TS-C2, Olympus Corporation) with a nominal spring constant of 100 pN/nm (25 kHz resonance frequency in water) were used. Circular coverglasses, 2.4 cm in diameter, were obtained from Menzel Gläser (Braunschweig, Germany). 3-Aminopropyl dimethyl ethoxysilane (APDMES) was purchased from ABCR GmbH (Karlsruhe, Germany). NHS-PEG-Maleimide ($5\,\mathrm{kDa}$) was purchased from Rapp Polymer (Tübingen, Germany). Immobilized TCEP Disulfide Reducing Gel was obtained from Thermo Scientific (Pittsburgh, PA). The following standard chemicals were obtained from Carl Roth (Karlsruhe, Germany) and used as received: tris(hydroxymethyl)aminomethane (TRIS, >99% p.a.), $CaCl_2$ (>99% p.a.), sodium borate (>99.8% p.a), NaCl (>99.5% p.a.), ethanol (>99% p.a.), and toluene (>99.5% p.a.). Borate buffer was 150 mM, pH 8.5. The measurement buffer for force spectroscopy was Tris-buffered saline (TBS, 25 mM TRIS, 75 mM NaCl, pH 7.2) supplemented with $CaCl_2$ to a final concentration of 1 mM. All buffers were filtered through a sterile $0.2\,\mu\mathrm{m}$ polyethersulfone membrane filter (Nalgene, Rochester, NY, USA) prior to use.

Protein Sequences

Sequences of protein constructs used in this work are listed here. Domains as well as engineered tags and residues are color-coded.

Xyn-XModDoc

Xylanase T129C
Linker or extra residues
X-module
Dockerin type III

```
M S H H H H H H K N A D S Y A K K P H I S A L N A P Q L D Q R Y K N E F T I G A
A V E P Y Q L Q N E K D V Q M L K R H F N S I V A E N V M K P I S I Q P E E G K
F N F E Q A D R I V K F A K A N G M D I R F H T L V W H S Q V P Q W F F L D K E
G K P M V N E C D P V K R E Q N K Q L L L K R L E T H I K T I V E R Y K D D I K
Y W D V V N E V V G D D G K L R N S P W Y Q I A G I D Y I K V A F Q A A R K Y G
G D N I K L Y M N D Y N T E V E P K R T A L Y N L V K Q L K E E G V P I D G I G
H Q S H I Q I G W P S E A E I E K T I N M F A A L G L D N Q I T E L D V S M Y G
W P P R A Y P T Y D A I P K Q K F L D Q A A R Y D R L F K L Y E K L S D K I S N
V T F W G I A D N H T W L D S R A D V Y Y D A N G N V V V D P N A P Y A K V E K
G K G K D A P F V F G P D Y K V K P A Y W A I I D H K V V P N T V T S A V K T Q
Y V E I E S V D G F Y F N T E D K F D T A Q I K K A V L H T V Y N E G Y T G D D
G V A V V L R E Y E S E P V D I T A E L T F G D A T P A N T Y K A V E N K F D Y
E I P V Y Y N N A T L K D A E G N D A T V T V Y I G L K G D T D L N N I V D G R
D A T A T L T Y Y A A T S T D G K D A T T V A L S P S T L V G G N P E S V Y D D
F S A F L S D V K V D A G K E L T R F A K K A E R L I D G R D A S S I L T F Y T
K S S V D Q Y K D M A A N E P N K L W D I V T G D A E E E
```

Coh-CBM C2A, C63S

CBM (C2A, C63S)
Linker or extra residues
CohIII
ybbR-Tag

M G T A L T D R G M T Y D L D P K D G S S A A T K P V L E V T K K V F D T A A D
A A G Q T V T V E F K V S G A E G K Y A T T G Y H I Y W D E R L E V V A T K T G
A Y A K K G A A L E D S S L A K A E N N G N G V F V A S G A D D D F G A D G V M
W T V E L K V P A D A K A G D V Y P I D V A Y Q W D P S K G D L F T D N K D S A
Q G K L M Q A Y F F T Q G I K S S S N P S T D E Y L V K A N A T Y A D G Y I A I
K A G E P G S V V P S T Q P V T T P P A T T K P P A T T I P P S D D P N A M A N
T P V S G N L K V E F Y N S N P S D T T N S I N P Q F K V T N T G S S A I D L S
K L T L R Y Y Y T V D G Q K D Q T F W S D H A A I I G S N G S Y N G I T S N V K
G T F V K M S S S T N N A D T Y L E I S F T G G T L E P G A H V Q I Q G R F A K
N D W S N Y T Q S N D Y S F K S A S Q F V E W D Q V T A Y L N G V L V W G K E P
G E L K L P R S R H H H H H H G S L E V L F Q G P D S L E F I A S K L A

Supplementary References

[1] Laskowski, R. A.; Hutchinson, E. G.; Michie, A. D.; Wallace, A. C.; Jones, M. L.; Thornton, J. M. PDBsum: a Web-based database of summaries and analyses of all PDB structures. *Trends in Biochemical Sciences* **22**, 488–490 (1997).

[2] Dietz, H.; Rief, M. Protein structure by mechanical triangulation. *Proceedings of the National Academy of Sciences* **103**, 1244–1247 (2006).

[3] Livadaru, L.; Netz, R. R.; Kreuzer, H. J. Stretching Response of Discrete Semiflexible Polymers. *Macromolecules* **36**, 3732–3744 (2003).

[4] Hugel, T.; Rief, M.; Seitz, M.; Gaub, H.; Netz, R. Highly Stretched Single Polymers: Atomic-Force-Microscope Experiments Versus Ab-Initio Theory. *Physical Review Letters* **94**, 048301 (2005).
